# Clinical decision support system RHINA in the diagnosis and treatment of acute or chronic rhinosinusitis

**DOI:** 10.1186/s12911-021-01599-3

**Published:** 2021-08-09

**Authors:** L. Hart, A. Polášková, P. Schalek

**Affiliations:** 1 Department of Otorhinolaryngology and Head and Neck Surgery, 3rd Faculty of Medicine and University Hospital Královské Vinohrady, Charles University in Prague, Prague, Czech Republic; 2 Charles University Computer Centre, 2nd Faculty of Medicine, Charles University in Prague, Prague, Czech Republic

**Keywords:** Clinical decision support system, Rhinosinusitis, Antibiotic resistance, EPOS

## Abstract

**Background:**

Rhinosinusitis is an inflammation of the sinonasal cavity which affects roughly one in seven people per year. Acute rhinosinusitis (ARS) is mostly, apart from allergic etiology, caused by a viral infection and, in some cases (30–50%), by a bacterial superinfection. Antibiotics, indicated only in rare cases according to EPOS guidelines, are nevertheless prescribed in more than 80% of ARS cases, which increases the resistant bacterial strains in the population.

**Methods:**

We have designed a clinical decision support system (CDSS), RHINA, based on a web application created in HTML 5, using JavaScript, jQuery, CCS3 and PHP scripting language. The presented CDSS RHINA helps general physicians to decide whether or not to prescribe antibiotics in patients with rhinosinusitis.

**Results:**

In a retrospective study of a total of 1465 patients with rhinosinusitis, the CDSS RHINA presented a 90.2% consistency with the diagnosis and treatment made by the ENT specialist.

**Conclusion:**

Patients assessed with the assistance of our CDSS RHINA would decrease the over-prescription of antibiotics, which in turn would help to reduce the bacterial resistance to the most commonly prescribed antibiotics.

**Supplementary Information:**

The online version contains supplementary material available at 10.1186/s12911-021-01599-3.

## Background

The term rhinosinusitis refers to an inflammation of the sinuses and nasal cavity which, according to histopathological findings and current imaging methods, occur simultaneously [1]. This disease of the upper respiratory tract affects roughly one in seven people per year and it has a significant impact on the quality of life and productivity of the individuals affected [2]. Thus, it can be concluded that acute rhinosinusitis (ARS) or chronic rhinosinusitis (CRS) is a widespread disease, with a large portion of the population impacted by either or both.

The cause of ARS, apart from an allergic etiology, is usually an infection. ARS is most commonly provoked by viruses such as Rhinovirus, Coronavirus, Influenza, Parainfluenza, Adenovirus, Respiratory Syncytial virus, as well as Enterovirus, which are able to induce a common cold [1]. In some cases (approximately 30–50%) the cause of sinonasal infections are bacteria, which secondarily populate the previous viral infection [3–5]. The most common of such bacteria are *S. pneumoniae*, *H. influenzae*, *S. aureus* and *M. catarrhalis* [6]. Despite these findings, ARS is commonly treated with antibiotics worldwide. Several studies demonstrated that antibiotics are prescribed in more than 80% of ARS cases [7, 8]. Differentiating diagnoses between viral and bacterial ARS has become a challenge, thus rendering the decision of physicians of whether to prescribe antibiotics more difficult [9].

In Europe, physicians should follow the guidelines which are summed up in the European Position-Paper on Rhinosinusitis and Nasal Polyps 2012 (EPOS 2012). According to these guidelines, it is recommended to consider antibiotic treatment of patients with ARS lasting more than 10 days or worsening after 5 days, if at least 3 of the following symptoms occur: colored secretion (one-sided predominance) and purulent nasal secretion, severe local pain (with one-sided predominance), fever more than 38 °C, an increased erythrocyte sedimentation/CRP, or a two-stage course of the disease (a deterioration after previous milder course) [1].

It has been observed that ARS will disappear in most patients without antibiotic treatment [3, 9–12]. Antibiotics (macrolides) are only recommended for treatment of CRS without polyps in patients with low overall IgE antibodies [1]. In other cases, systemic antibiotic treatment of CRS does not have any significant effect [13]. According to the National Ambulatory Medical Care in the USA, rhinosinusitis is the fifth most common diagnosis for which antibiotics are prescribed despite many randomized studies and clinical guidelines that question the benefits of antibiotics in moderate or uncomplicated ARS [3, 9–12]. A recent multi-national study highlighted a problem of antibiotic misuse by physicians [14]. A randomized double-blind study has identified that if the oral streptococcal microbiome in healthy individuals is exposed to azithromycin and clarithromycin, it is the main driving force of antibiotic resistance [15]. Systemic over-usage of antibiotics might cause many side effects, but also leads to an increase in antibiotic resistance [16–18] with potential global consequences and thus poses a threat to the health of the general population [19]. An appropriate tool in medical practice, one which would make the decision-making process more precise to determine whether there is a need of use of antibiotics in treatment of sinonasal infections, would validly contribute to reduce the excessive overconsumption of antibiotics worldwide [8, 20].

In our study we design and prepare specific methods for objective examination of patients suffering from one of the forms of rhinosinusitis. The examination, diagnosis and treatment of these patients are usually carried out in ENT (ear, nose, throat) departments by an ENT specialist and thus in accordance with valid EPOS recommendations [8]. However, a large proportion of patients are also examined, diagnosed and treated in the primary medical sphere (general practitioners), who show generally lower adherence to valid recommendations [21]. Diagnosis and treatment of some patients are not always pursuant to generally accepted guidelines. It can be assumed that if valid recommendations are presented electronically to these treatment providers, the adherence of these doctors to valid recommendations will be increased, thus allowing them to make “safer” decisions in the treatment of patients [22]. The aim of this study was to create a CDSS for examination, diagnosis and treatment of a patient with rhinosinusitis, based on valid EPOS recommendations and available to all physicians, specialists and practitioners who encounter a patient reporting symptoms of rhinosinusitis. The purpose of this article is not only to present the software we have developed for better treatment of patients with rhinosinusitis, but also to introduce the terminology of expert systems to the health reader, as well as to introduce the history of this artificial intelligence in medicine and to point out its advantages and disadvantages in common medical practice.

The application of expert systems gained traction during the 70 s and 80 s, when these systems were perceived as being based on cutting-edge knowledge received from the top experts. It was clear that the quality of these systems depended much more on the quality of knowledge than on the quality of the mechanism for their use. To date, thousands of systems have emerged covering a wide variety of issues. At least half of them focus on the field of medicine, most likely because medical knowledge is very well structured. From the beginning of their development, numerous software systems have been introduced: Mycin—diagnosis and treatment of bacterial infections in hospitalized patients [23], internal medicine systems—Internist-1 [24] and QMR [25], pneumology PUFF [26], oncology—Oncocin [27] etc., and in the ENT area, for example, the expert system for disorders of the equilibrium system [28] or allergic rhinitis [29, 30].

CDSS are designed to standardize clinician decision-making about individual patients at the point in time that these decisions are made [31]. Since the publication of “To Err is Human” [32], CDSS in conjunction with CPOE (Computer Based Order Entry) have been identified as key systems for assisting in the prevention of medical errors and promoting patient safety [33, 34]. CDSS can be categorized as knowledge-based systems and non-knowledge-based systems. Knowledge based CDSS simulates a person's thinking, offering information to the user and thus assisting him in his own decision making process [31]. Such a system consists of three basic parts: (1) the knowledge base, (2) the inference engine and (3) the communication mechanism. The knowledge database is composed of compiled information in the form of if–then rules, probabilistic interactions of symptoms with diagnosis, etc. [31]. The second part is the inference engine or reasoning mechanism, which consists of formulas for combining rules and associations from the knowledge base with actual patient data. The third inseparable part is the mechanism of communication, i.e. how the patient's data gets into the system and the output of the system to the user who makes the decision [31]. The inference engine confronts patient’s information (symptoms) with its knowledge base and offers the user a possible diagnosis for these symptoms. Since the physician knows the patient more deeply, and all patient data is not able to be reported in any CDSS, it is therefore up to the physician to make the last correction in terms of avoiding unlikely diagnoses [35].

Non-knowledge systems utilize a form of artificial intelligence called machine learning that allows a computer to learn from past experience and / or recognize patterns in medical data [36]. These are either artificial neural networks (ANNs) that simulate human thinking and learn from examples, or genetic algorithms based on the evolutionary theory of Darwin, which speaks of natural selection and survival of the most capable individuals [37].

A study found that CDSS improves physician´s performance in 62 out of 97 cases, or roughly 64% of the time [38]. Despite this, most doctors are reluctant to cooperate with the CDSS in their practice as they consider their recommendation process opaque [39]. The same reluctance of physicians to work with electronic forms of health information was confirmed by the Cochrane review [40].

Nevertheless, Giarratamo et al*.* lists many of the benefits these systems provide to their users, such as increased availability, reduced costs, durability, multiplicity, high objectivity, and fast response [41].

## Implementation

**Fig. 1:**
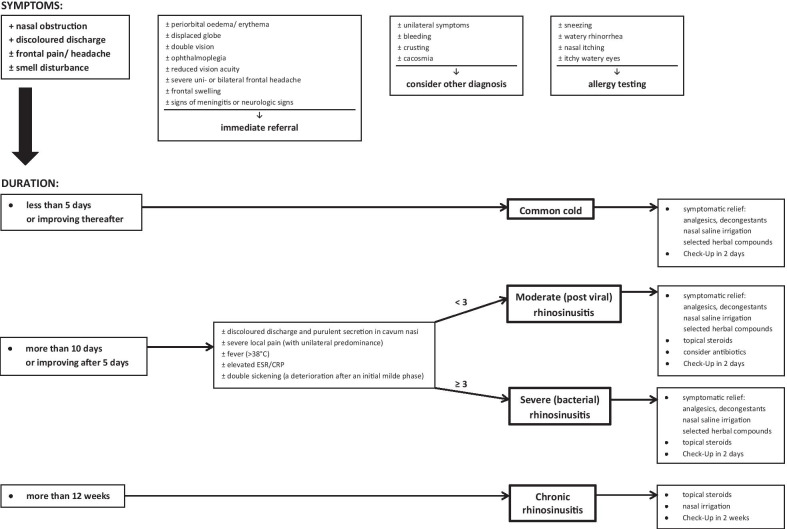
Algorithm of the system

We have developed a clinical decision support system, RHINA, based on a web application and therefore it is universally available online. The CDSS RHINA helps the physician in making decisions during diagnosis and therapy of patients with an acute or chronic rhinosinusitis (Fig. 1). The knowledge base strictly follows valid EPOS recommendations and the inference engine consists of direct chain logic formulas and “if–then” rules. The inference engine faces input data (symptoms) with its knowledge base and presents the user a diagnosis with its therapeutic recommendation. The application was developed in PHP scripting language and MySQL database engine for data storage. The web interface of the application is created in HTML 5, as specified by World Wide Web Consortium, and it uses JavaScript and jQuery (JavaScript library), CCS3 (cascading style sheets) and PHP scripting language (Additional file 1: Fig. S1).

This application is compatible with all major browsers, except Internet Explorer 10 and older versions. Therefore, we have created a widely accessible platform-independent web application.

An internet browser sends a request to a web server and the server returns the requested data (HTML page, CSS styles and JavaScript code) back to the user. The application uses jQuery library for real-time remote analysis on the web server processed by PHP scripts evaluating user inputs and solving the problem with the decision tree model (Additional file 1: Fig. S2).

## Results

Our study population consists of 1465 patients (aged 18–87) who sought care at the Department of Otorhinolaryngology at the Faculty Hospital Královské Vinohrady in Prague in 2016, 4 years after publishing of the EPOS. The selection was based on the international diagnosis codes (ICD) and the study included *622 patients with ARS* and *843 patients with CRS* symptoms.

In patients with acute symptoms (less than 12 weeks), symptomatic decongestive therapy was indicated in *53.9%* of patients (335 patients—common cold), enriched with intranasal corticoid in *29.9%* (186 patients—postviral rhinosinusitis) and antibiotics were prescribed to *16.2%* of patients (101 patients—purulent rhinosinusitis) (Additional file 1: Fig. S3).

In instances of CRS (more than 12 weeks), for *98.5%* of patients (830 patients) the drug of choice was intranasal corticosteroid and antibiotics were prescribed in *1.5%* of cases (13 patients), most often postoperative purulent complication, or a therapeutic trial of macrolide treatment in refractory, polyp-free chronic rhinosinusitis in accordance with recent studies, e.g. Cochrane Review [13] (Additional file 1: Fig. S4).

Coming to a conclusion, out of all patients in our study population (1465) only *7.78%* (114 patients) were treated with antibiotics according to EPOS guidelines (Fig. 1).

We manually utilized the CDSS RHINA on an above-mentioned study population (1465 records) in retrospective study in order to determine the consistency of the presented diagnostic tool. The CDSS RHINA was consistent in 90.2% of cases.

The ENT specialist, unlike our CDSS, indicated antibiotics in patients with chronic immunosuppressive therapy (nephrotic syndrome, Wegener's vasculitis, refractory psoriasis, chronic obstructive pulmonary disease, multiple sclerosis, and rheumatoid arthritis) and anticancer treatment (chronic myeloid leukemia and lymphoma). Furthermore, in other patients during the search for the focus of infection, prior to stem cell transplantation and aortic valve implantation, antibiotics have also been used based on the experience of physicians in recidivists with a pathological ostiomeatal unit (concha bullosa, deviation of the septum). The last “CDSS error” was a patient with another metachronous purulent infection requiring antibiotics—tonsillitis and acute mesotitis. This result faithfully demonstrates that the CDSS drew on its knowledge base in diagnostic and therapeutic decision-making and did not take into account other information such as a patient's overall health as it lacked the necessary knowledge and experience of a human physician. Nevertheless, we see its benefit mainly in primary care, outpatient clinics, where it could assist in diagnosis and treatment of immunocompetent patients presenting with symptoms of rhinosinusitis of any duration, and so contribute to a significant reduction in antibiotic over-prescription, which in turn limits the growth of resistant bacterial strains in the epidemiological group.

## Discussion

CDSSs help improve the quality of practitioners' knowledge, reduce decision-making conflicts and thereby improve patient-doctor communication [42, 43].

Sim et al*.* encourage the use of CDSS to promote the practice of evidence-based medicine, provided that high-knowledge information is introduced into the knowledge base of the CDSS [44]. New nosological units or subunits are emerging every day, hundreds of new medicines flood the pharmaceutical market every month. The knowledge base of each CDSS must therefore be regularly updated and include uptodate information. Who is to blame if the end-user makes a medically erroneous decision based on outdated knowledge-based information? Scientists addressed this issue several decades ago [45]. Ultimately it can be argued that the overall benefits of the CDSS are outweighed over the drawbacks of outdated knowledge-based information. CDSS has already demonstrated its benefit at the pharmaceutical level, reducing the frequency of adverse drug-drug interactions and preventing excessive or under-prescription of drugs [46, 47]. With the demand of health management for the best possible health care at the lowest cost, the interest in the use of CDSS in clinical practice is growing and their future is promising [48]. Similarly, as fear of using computers in clinical practice has subsided, the opposition to the widespread application of CDSS as part of contemporary medicine is expected to subside. If the new CDSS are sufficiently accessible and user-friendly, the medical community may be more interested in this phenomenon. Some even believe that the computer will replace the human doctor [49]. However, we must never forget that artificial intelligence should only support a person and strengthen their decision-making process, which, after all, is the ultimate responsibility of the physician.

## Conclusion

Our CDSS RHINA supports and facilitates the work of general practitioners in the diagnosis and treatment of rhinosinusitis patients, while remaining in line with the current EPOS recommendations. In general practice, general practitioners use computer technology and the implementation of this software into equipment already purchased will not increase the cost of office operation. Furthermore, the cost of usage of the CDSS application can be reduced and its accessibility increased in low income communities through mobile phone-based applications, which is the aim of our future study. The CDSS RHINA was subjected to extensive testing in the form of a retrospective study of a total of 1465 patients with varying degrees of rhinosinusitis, in 90.2% of cases it coincided with the diagnosis and treatment determined by the ENT specialist. Thus, it can be assumed that patients will be treated by using our CDSS RHINA with a higher adherence to valid EPOS recommendations and that over-prescription of antibiotics in a given epidemiological group will be reduced with the hope of reducing resistant bacterial strains to the most commonly prescribed antibiotics.

Because we want keep our CDSS RHINA up to date, we are recently working on an implementation of the new European Position Paper on Rhinosinusitis and Nasal Polyps from 2020. Once the knowledge base will be revised, the update will be completed.

## Supplementary Information

**Additional file 1.** **Figure S1**: Structure of CDSS RHINA. **Figure S2**: Decision making process in CDSS RHINA. **Figure S3**: Study population of patients with ARS. **Figure S4**: Study population of patients with CRS

## Data Availability

All data and materials are available and might be requested from the main author: libor.hart@gmail.com. Project name: RHINA; Project home page: http://195.113.37.218/~sasa/ryma/evidences3; Operating system: platform independent; Programming language: PHP, MySQL; Other requirements: none; License: GNU GPL; Any restrictions to use by non-academics: no license needed.

## Abbreviations

ANN: Artificial neural networks
ARS: Acute rhinosinusitis
CCS 3: Cascading style sheets 3
CDSS: Clinical decision support system
CPOE: Computer based order entry
CRP: C reactive protein
CRS: Chronic rhinosinusitis
EPOS: European Position-Paper on Rhinosinusitis and Nasal Polyps
ENT: Ear, nose, throat
GNU GPL: GNU General Public License
HTML 5: Hypertext Markup Language 5
IgE: Immunoglobulin E
jQuery: Java Query
MySQL: My Structured Query Language
PHP: Hypertext preprocessor
PUFF: An expert system for interpretation of pulmonary function data
QMR: Quick Medical Reference
USA: United States of America
