# Supplementary material for: Clinical decision support system RHINA in the diagnosis and treatment of acute or chronic rhinosinusitis
Source: BMC Med Inform Decis Mak. 2021 Aug 9;21:239. doi: 10.1186/s12911-021-01599-3 (PMC8350307; doi:10.1186/s12911-021-01599-3)

SUPPLEMENTAL MATERIAL

SUPPLEMENTAL FIGURE 1:

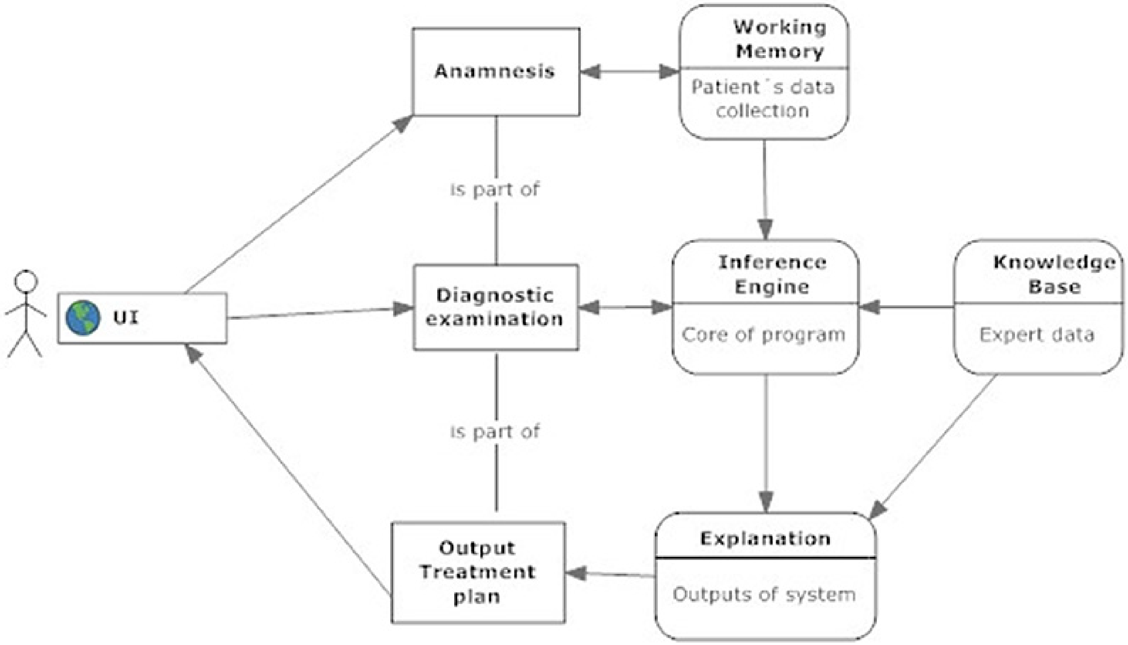

SUPPLEMENTAL FIGURE 2:

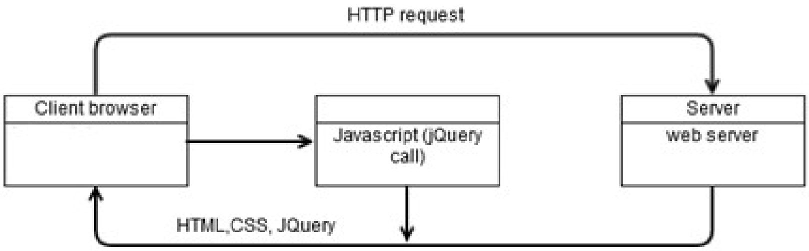

Sup. Fig 1: Decision making process in CDSS RHINA

SUPPLEMENTAL FIGURE 3:

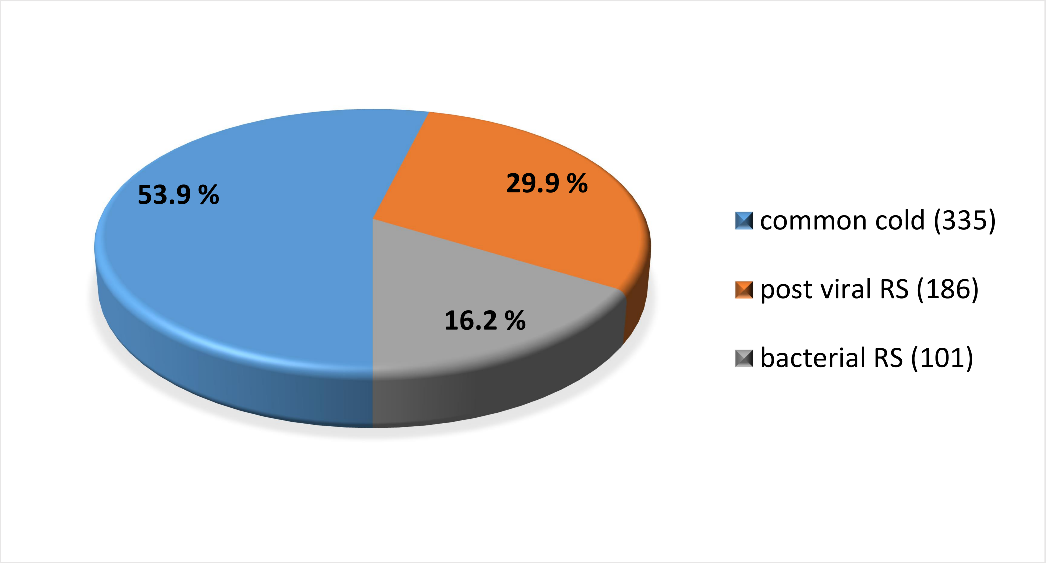

SUPPLEMENTAL FIGURE 4:

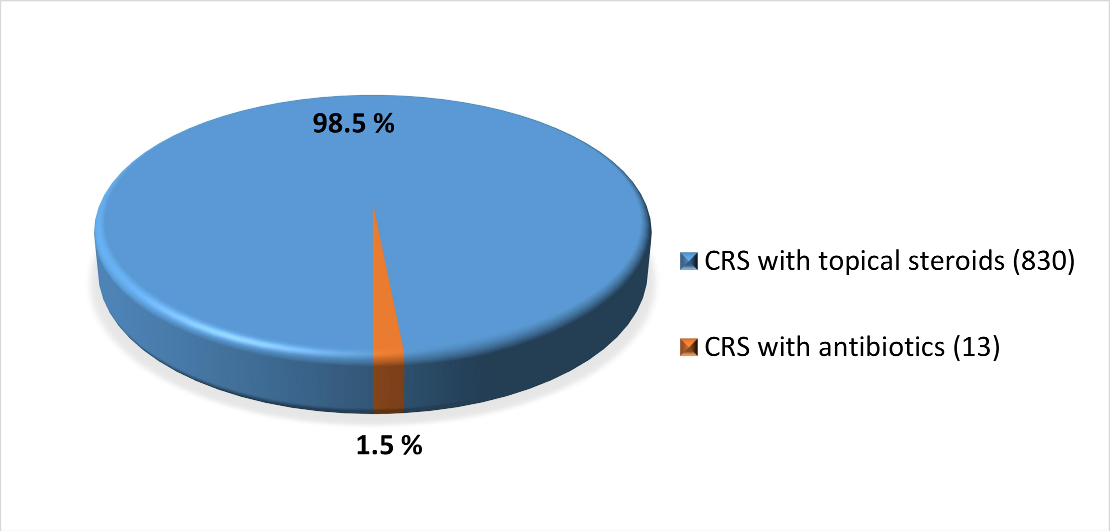

Supplement: Supplementary file 1 — Additional file 1. Figure S1: Structure of CDSS RHINA. Figure S2: Decision making process in CDSS RHINA. Figure S3: Study population of patients with ARS. Figure S4: Study population of patients with CRS [file 12911_2021_1599_MOESM1_ESM.pdf]
